# New World screwworm (*Cochliomyia hominivorax*) myiasis in feral swine of Uruguay: One Health and transboundary disease implications

**DOI:** 10.1186/s13071-020-04499-z

**Published:** 2021-01-07

**Authors:** Martín Altuna, Paul V. Hickner, Gustavo Castro, Santiago Mirazo, Adalberto A. Pérez de León, Alex P. Arp

**Affiliations:** 1 Universidad de la República, Grupo Proyecto Jabalí, Montevideo, Uruguay; 2 Knipling-Bushland U.S. Livestock Insects Research Laboratory and Veterinary Pest Genomics Center, U.S.Department of Agriculture-Agricultural Research Service (USDA-ARS), Kerrville, TX USA

**Keywords:** Screwworm, Myiasis, *Cochliomyia hominivorax*, Invasive species, One Health, Zoonosis, Transboundary, Feral swine, *Sus scrofa*

## Abstract

**Background:**

Feral swine (*Sus scrofa*) are highly invasive and threaten animal and human health in the Americas. The screwworm (*Cochliomyia hominivorax*) is listed by the World Organization for Animal Health as a notifiable infestation because myiasis cases affect livestock, wildlife, and humans in endemic areas, and outbreaks can have major socioeconomic consequences in regions where the screwworm has been eradicated. However, a knowledge gap exists on screwworm infestation of feral swine in South America, where the screwworm is endemic. Here, we report screwworm infestation of feral swine harvested in Artigas Department (Uruguay), where the Republic of Uruguay shares borders with Brazil and Argentina.

**Methods:**

Myiasis caused by the larvae of screwworm were identified in feral swine with the support and collaboration of members of a local feral swine hunting club over a 3-year period in the Department of Artigas. Harvested feral swine were examined for the presence of lesions where maggots causing the myiasis could be sampled and processed for taxonomic identification. The sites of myiasis on the body of infested feral swine and geospatial data for each case were recorded. The sex and relative size of each feral swine were also recorded. Temperature and precipitation profiles for the region were obtained from public sources.

**Results:**

Myiases caused by screwworms were recorded in 27 of 618 the feral swine harvested. Cases detected in males weighing > 40 kg were associated with wounds that, due to their location, were likely caused by aggressive dominance behavior between adult males. The overall prevalence of screwworm infestation in the harvested feral swine was associated with ambient temperature, but not precipitation. Case numbers peaked in the warmer spring and summer months.

**Conclusions:**

This is the first report on myiasis in feral swine caused by screwworm in South America. In contrast to myiasis in cattle, which can reach deep into host tissues, screwworms in feral swine tended to cause superficial infestation. The presence of feral swine in screwworm endemic areas represents a challenge to screwworm management in those areas. Screwworm populations maintained by feral swine may contribute to human cases in rural areas of Uruguay, which highlights the importance of the One Health approach to the study of this invasive host species–ectoparasite interaction.
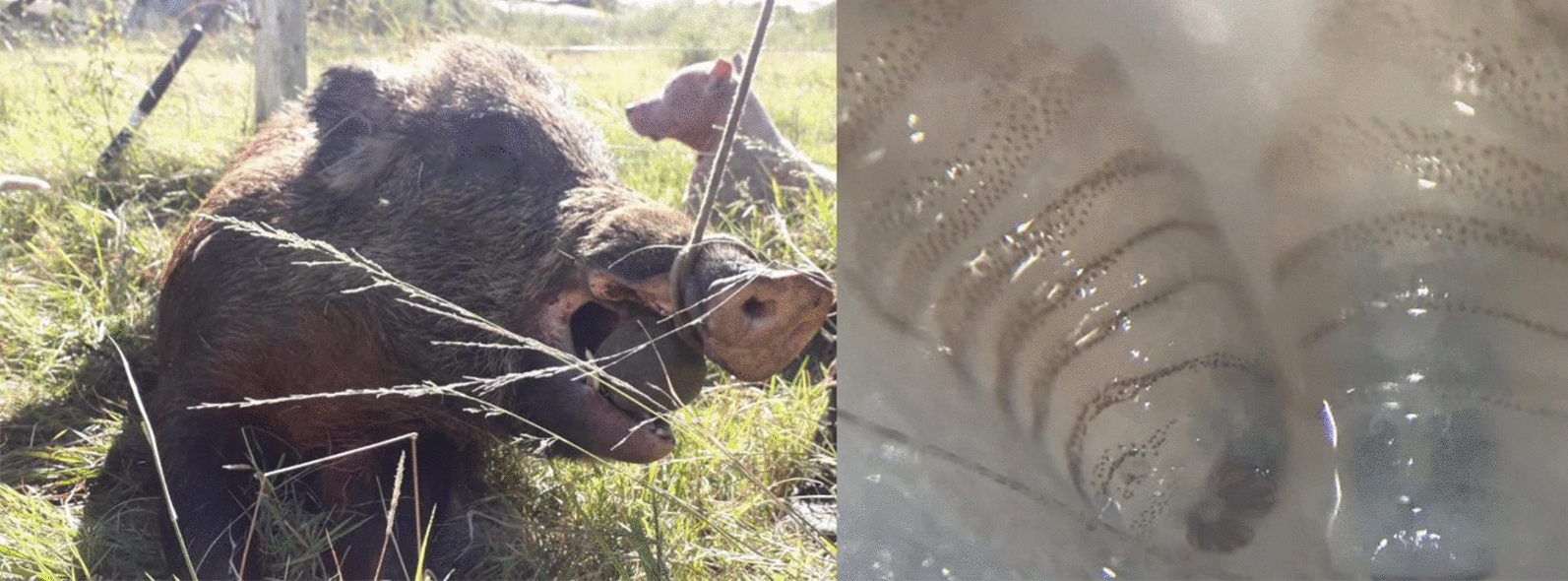

## Background

Interactions between invasive wildlife species and high-consequence zoonotic parasites or vectors can have One Health and transboundary disease implications. Multiplier effects from these interactions can exacerbate the risks to the health of human and domestic and wild animal populations, as well as the environment [1, 2]. One such interaction that has not been explored is that between feral swine (*Sus scrofa*) and screwworm (*Cochliomyia hominivorax*). Feral swine are highly invasive and threaten animal and human public health because they host parasites and vectors of zoonotic importance in the Americas [3]. Screwworm is listed by the World Organization for Animal Health as a notifiable infestation because myiasis cases affect livestock and other domestic animal species, wildlife, and humans in endemic areas [4]. Screwworm outbreaks can be of high socioeconomic consequence in regions of the Americas where this ectoparasite of warm-blooded animals has been eradicated [5, 6]. Although feral swine were known to be infested before the screwworm was eradicated from North and Central America [7], a knowledge gap exists for this invasive species in South America regarding cases of myiasis caused by screwworm.

Screwworms were originally described infesting humans in 1858 [8]. Today this parasite remains endemic in South America, where they cause myiasis, which is regarded as a neglected zoonosis. Screwworm affects domestic animals and wildlife and inflict significant loss to livestock producers [9, 10]. Similar conditions in the past triggered research in the USA that translated into the development the sterile insect technique (SIT), which was applied areawide to eventually eliminate screwworms from North and Central America, and Puerto Rico [11]. Biotechnology-based SIT approaches are under development for sustainable operations in the screwworm barrier zone maintained on the border between Panama and Colombia through bilateral collaboration between the governments of Panama and the USA [12, 13]. Screwworm control efforts related to animal health and production in countries of South America include monitoring and treatment of myiasis cases using insecticides when livestock are susceptible to infestation, such as in newborn navels, castrations, and other practices related to herd management and traceability [14]. In temperate regions of South America, additional protection from infestation can be achieved by planning livestock births, branding, castrations, or sheering to occur in months with lower screwworm abundance [15]. Surveillance in livestock is a key component of screwworm control, but monitoring and reporting of myiasis cases in wildlife is seldom practiced.

Wildlife involvement in endemic areas maintains screwworm populations that also affect humans and livestock. Prior to eradication the USA, it was estimated that up to 2–3% of wild animals could be infested with screwworms in endemic regions [16]. White-tailed deer (*Odocoileus virginianus*) die-offs in parts of the USA were associated with myiasis before screwworm eradication [17, 18]. It has also been argued that the disappearance of the screwworm as a natural wildlife population control is part of the reason why white-tailed deer populations have increased markedly in those parts of the USA [17]. In the 1950s, screwworm infestations were reported in feral swine in the state of Florida (USA), and control of the swine populations was considered a priority at that time as a means to reduce the screwworm incidence in deer herds [7]. The importance of wildlife as a host for screwworm was highlighted during the outbreak in the Florida Keys (USA) in 2016, which resulted in the death of 135 endangered Key deer (*Odocoileus virginianus clavium*) [6]. Therefore, surveillance for myiasis cases in wildlife species, including invasive feral swine, could enhance the efficiency of areawide screwworm management programs [19].

In 1982, feral swine were officially declared a national pest in Uruguay after they were introduced to the country at the beginning of the last century for hunting purposes [20]. Females are capable of producing up to two litters of four to five piglets each year that can reach sexual maturity in less than 1 year, a reproductive output fourfold greater than white-tailed deer [21]. As is the case in other parts of their invaded range, in Uruguay, feral swine also cause extensive damage to agriculture, critical infrastructure, and private property, as well as being a public safety hazard [22, 23]. Organized hunting in Uruguay helps control feral swine and under the proper sanitary conditions provides economic opportunities to hunters and farmers. Research on the causes of myiasis in feral swine was listed as part of the activities needed to inform plans to develop a screwworm control program in Uruguay [24].

This study is the result of a public–private partnership involving an interdisciplinary project on feral swine led by the Colleges of Sciences and Veterinary Medicine of the University of the Republic of Uruguay, in collaboration with the National Association of Hunters in Uruguay, and other regional feral swine hunting and control associations referred to as ProJAB (its acronym in Spanish). In particular, myiasis cases in feral swine reported by members of the Association for the Control of Feral Swine (ACJA) in the Province of Artigas (Uruguay) provided the opportunity to investigate if screwworms were involved. The support and involvement of hunters and associated groups proved to be invaluable to obtain data on screwworm cases in feral swine of Uruguay and stresses the high impact that citizen science can have on pest management programs [25, 26].

## Methods

**Fig. 1:**
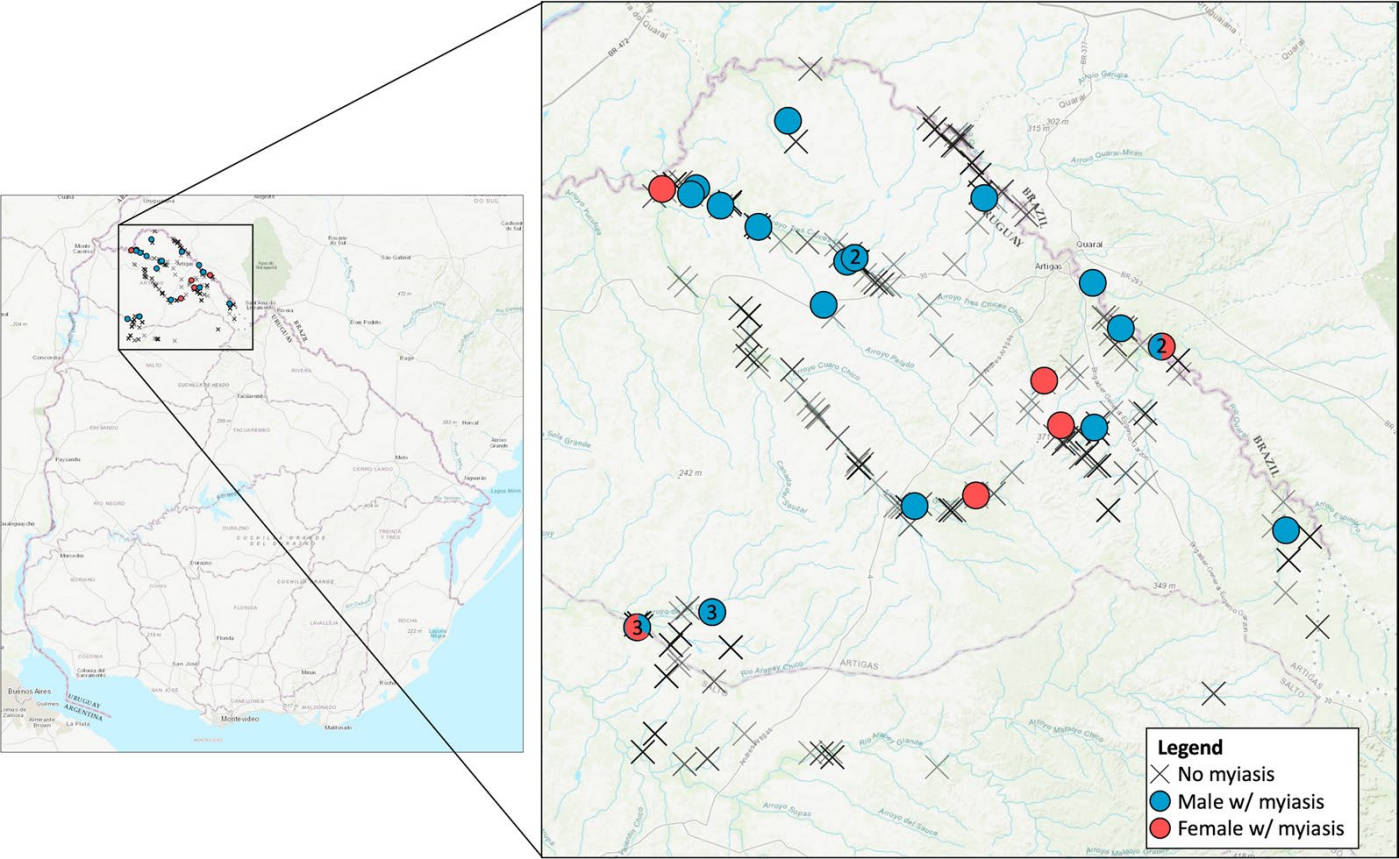
Map of Uruguay (left) with the sampling area in the departments of Artigas and Salto along the Brazilian border expanded (right). Locations of feral swine (*Sus scrofa*) capture are shown (see legend), with sites marked as swine having or not having myiasis. Darker X‘s indicate multiple swine sampled at that site. Sites with more than one myiasis case have the total number of cases given in the circle. Sex ratio is shown as a pie chart

Feral swine were harvested over a 3-year period (May 2017 to April 2020) by members of the ACJA in the Artigas and northern Salto departments of Uruguay (Fig. 1; total sampling data is available in Additional file 1: Dataset S1). Artigas department is located in the northern-most portion of Uruguay. The western portion of the department is alluvial plain, while the central and eastern parts are hill ranges. The climate is sub-tropical with an average monthly temperature of 19 °C and an average total annual rainfall of 1400 mm. The primary economic activity is raising sheep and cattle.

**Fig. 2:**
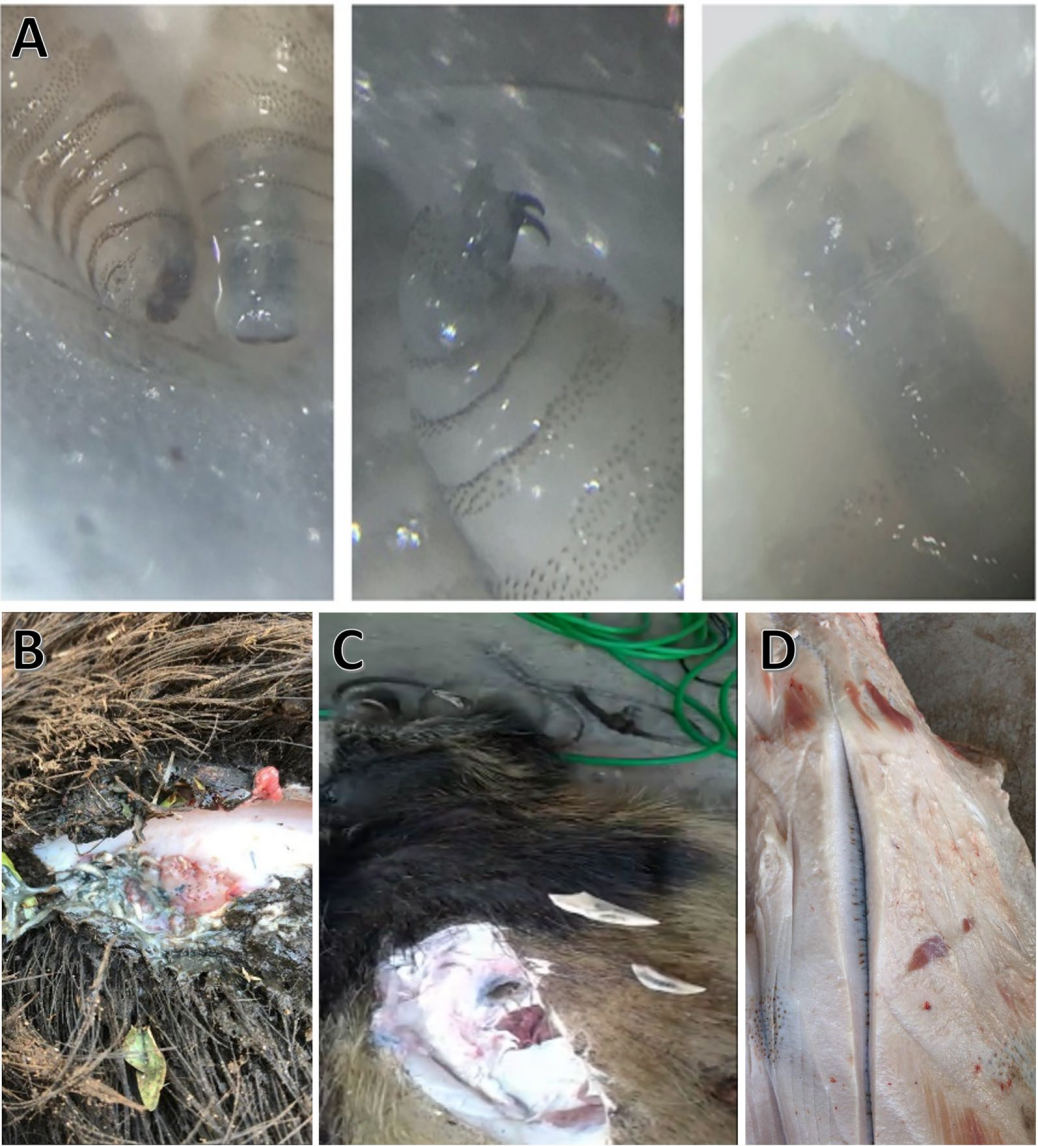
Images of representative screwworm larvae samples (**a**, top row), myiasis of a feral swine (**b**), male shoulder shield that had the tusks of another male broken off and embedded (**c**), and shoulder shield from a large male split to show the thickness (approximately 5–6 cm) (**d**)

The hunters used several methods to harvest the feral swine, including dogs, firearms, and cage traps. Upon capture, the feral swine were euthanized, geo-referenced, and inspected to assess the general state of health and the presence of parasites (ticks, lice, dipteran larvae) as part of a comprehensive study to assess zoonosis. Size and sex were recorded for all animals, with size being categorized as < 20, 20–40 or > 40 kg (large). Harvested feral swine were inspected visually to identify wounds, which were then checked for myiasis. Upon the detection of myiasis, the site of larval infestation, larval instar, and probable cause of wound were also recorded. Probable cause of the wound was based on the location and shape of the wound and determined by the hunters who have extensive experience hunting feral swine. All larvae present were collected with a stainless tweezer and stored in 70% ethanol for transport to the laboratory where they were identified to species using a stereoscope (4× and 10× magnification). Identification of *C. hominivorax* larvae was based on tracheole color, spine structure and distribution, and oral hooks [27] (Fig. 2a).

The map of hunted feral swine and myiases cases was created using the ArcGIS online application. Monthly average temperature and total precipitation data was obtained from the Artigas department meteorological station (http://www.inumet.gub.uy) and accessed through Weather Underground (www.wunderground.com); this information is provided in Additional file 2: Dataset 2. Statistical analysis was conducted in the R statistical environment (v3.5.1; [28]). The Fisher’s exact test was used to test for nonrandom associations between sex and size with myiasis. Linear regressions were used to determine correlations between myiases and temperature and precipitation. Differences in hunting pressure by month was conducted using analysis of variance.

## Results

A total of 618 feral swine were examined during the study period, of which 27 were infested with dipteran larvae (Fig. 1; Table 1). Animal size, sex, location of capture, and area of body infested for the 27 feral swine with myiasis is reported in Additional file 3: Dataset 3. Microscopic examination of the larvae revealed all cases of myiasis were due to infestation by *C. hominivorax*. The number of infested males was significantly greater than expected by random chance (*P* = 0.003), with 20 of 282 males with screwworm infestation compared to seven of 336 females. In addition, the number of large adults with screwworm infestation was greater than expected (*P* < 0.001), with 26 of 332 large adults having screwworm infestation compared to only one of 286 small/medium swine. These data suggest that there is a greater prevalence of screwworm infestation in large adults, especially large adult males.

**Table 1:** Size, sex, and number of feral swine (*Sus scrofa*) examined in the study

Size and sex	No. examined	No. with myiasis
Females (> 40 kg)	167	7
Males (> 40 kg)	165	19
Females (20–40 kg)	122	0
Males (20–40 kg)	71	0
Females (< 20 kg)	47	0
Males (< 20 kg)	46	1

Myiasis was found in 12 areas broadly distributed across the body of the swine (Table 2). Despite these infestations, the feral swine showed no signs of severe morbidity due to the myiasis and were otherwise healthy. In some cases, the site of myiasis was covered in mud to a greater extent than the rest of the body, suggesting the possibility that their wallowing behavior could provide some protection by limiting the severity of infestation.

**Table 2:** Locations of myiasis on the feral swine body

Myiasis site	Male	Female	Total
Shoulder	4 (20%)	1 (14%)	5 (19%)
Loin	2 (10%)	1 (14%)	3 (11%)
Eye	1 (5%)	0 (0%)	1 (4%)
Nose	1 (5%)	0 (0%)	1 (4%)
Head	2 (10%)	0 (0%)	2 (7%)
Umbilical	1 (5%)	0 (0%)	1 (4%)
Neck	2 (10%)	0 (0%)	2 (7%)
Leg	0 (0%)	1 (14%)	1 (4%)
Hind Leg	1 (5%)	0 (0%)	1 (4%)
Flank	1 (5%)	2 (29%)	3 (11%)
Genitals	1 (5%)	1 (14%)	2 (7%)
Ribs	3 (15%)	0 (0%)	3 (11%)

Values in table are presented as the number of infestations at each part of the body, with the percentage in parentheses

The hunters participating in this study proposed sources of wounds resulting in myiasis (Table 3). Across all sampled swine, it was concluded that most of the wounds detected were the result of intraspecies aggression (15/27), primarily between males (13/0). The second leading cause of myiasis was non-lethal bullet wounds (5/27). The only juvenile pig collected with myiasis had the infection in the umbilical region.

**Table 3:** Suspected sources of wounds resulting in myiasis

Wound Source	Male	Female	Total
Bullet	3 (15.0%)	2 (28.6%)	5 (18.5%)
Fight between feral swine	13 (65.0%)	2 (28.6%)	15 (55.6%)
Fight with dogs	0 (0.0%)	2 (28.6%)	2 (7.4%)
Umbilical	1 (5.0%)	0 (0.0%)	1 (3.7%)
Vegetation	1 (5.0%)	1 (14.3%)	2 (7.4%)
Barbed wire	2 (10.0%)	0 (0.0%)	2 (7.4%)

Values in table are presented as the number of different wound sources, with the percentage in parentheses

**Fig. 3:**
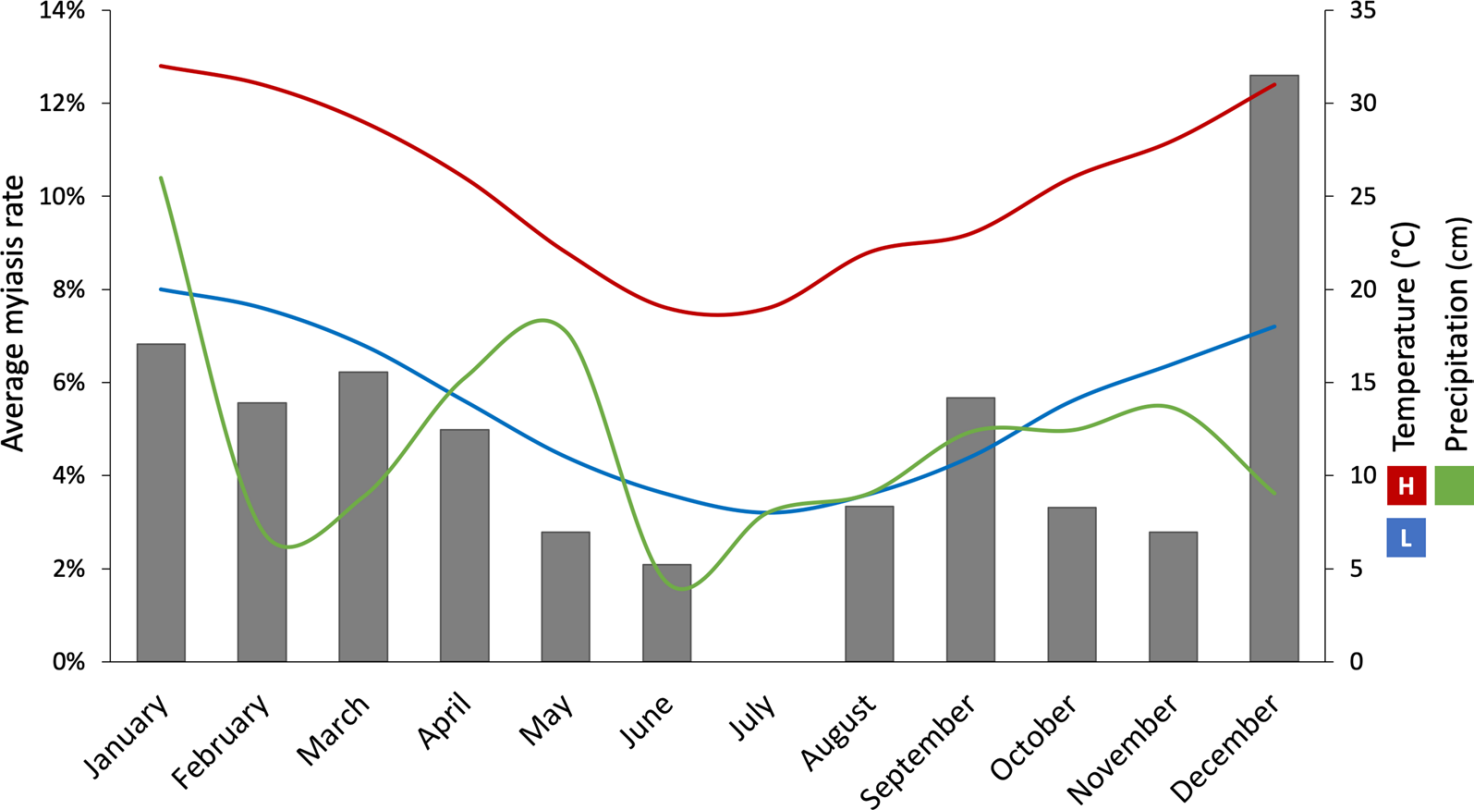
Average rate of myiases in harvested feral swine presented as bars with scale on the left vertical axis. Average monthly high and low temperatures and average total precipitation during the study period are presented on the right vertical axis

The average percentage of feral swine harvested with myiases reported is shown in Fig. 3. The number of harvested feral swine was consistent between months (*F*_(11,25)_ = 1.48, *P* = 0.20), with an average (± standard deviation) of 16.73 ± 8.81 feral swine harvested per month. The number of myiases reported were not correlated to the number of harvested feral swine (*R*^2^ = 0.090, *P *= 0.179), indicating sampling depth was able to record accurate myiasis rates. Cases of myiasis were highest in the spring months September to December (6.71% feral swine with myiasis), and lowest in the winter months July and August (1.09% feral swine with myiasis). The highest percentage of myiasis cases were reported in December (12.9%) and the lowest in June (no cases). Total monthly myiasis cases were correlated to monthly average temperature, with the number of cases increasing with increasing average monthly high (*R*^2^ = 0.438, *P* < 0.001) and low (*R*^2^ = 0.102, *P* = 0.031) temperatures. Total monthly precipitation did not correlate to the presence of myiases (*R*^2^ = 0.004, *P* = 0.289).

## Discussion

To the authors’ knowledge, this is the first epidemiological report of screwworm myiasis in feral swine from South America. The myiasis cases in feral swine were reported by hunters in the Artigas and Salto provinces of Uruguay between April 2017 and April 2020. Of the 618 swine examined, 27 were infested with maggots. The screwworm infestation prevalence (4.36%) recorded in the feral swine captured in this study is relatively higher than the estimated 2–3% in wildlife reported by Lindquist in 1937 in Texas [16], and lower than the > 5% recorded in domestic pigs in 1987 in Yucatan, Mexico [29]. The prevalence reported here is similar to that reported previously in sheep (5.7%) and cattle (3.4%) in Uruguay [24].

Screwworm cases were detected primarily in large male feral swine, and primarily in wounds that the hunters suspected as being the result of fighting between males. Dominance behavior in swine includes the males lining up facing each other and pushing at the shoulders, which can leave large lacerations due to their tusks, and biting the neck, ears, and face (Fig. 2b) [30]. These opportunities to lay eggs were exploited by screwworm female flies because 41% of the myiases occurred in those body parts of the host. Wound infestation in males associated with lesions resulting from feral swine fighting was also reported to be common in this wild host before screwworm eradication in Florida was accomplished [7].

Genital myiases in post-birthing females and in juvenile navels are very common in livestock and a primary concern to ranchers [31, 32]. Of the feral swine harvested in this study, only one female with genital myiasis and one juvenile male with navel myiasis were observed. It is possible that in feral swine these are not common sources of myiases, or that these were not commonly observed because they lead to mortality. Navel myiases may have also been underreported, as more adult animals were harvested.

Hunters who participated in this study reported that although myiases are common in feral swine, they did not observe myiases causing serious morbidity or mortality (see Fig. 2b for a typical myiasis in feral swine). Physically, feral swine have thick skin that could inhibit the formation of myiasis and be inhospitable to screwworm larvae. Additionally, male swine develop thick layers of skin and cartilage near the shoulder, called shields [20], which protect males during fights for dominance and may also inhibit screwworm development (Fig. 2c, d). Feral swine behavior may also prevent or act as a treatment of myiases. Wallowing has many benefits to the animal, including thermal regulation, ultraviolet-light protection, and protection against ectoparasites and biting flies [33]. Screwworm larvae in a myiasis are surrounded by fluid, but they must be exposed to air through their terminal spiracles; thus, coating a myiasis in mud or water could suffocate larvae [34]. Coating a wound in mud could also prevent the release of odors that attract gravid female screwworm flies to a wound and stimulate oviposition. Feral swine also exhibit a rubbing behavior associated with wallowing that could remove unhatched egg masses or larvae close to the surface. They also soak or swim in water, behaviors that have been observed in deer to help clean myiases [16]. Thus, it is possible feral swine also intentionally soak to remove screwworm larvae.

Feral swine that survive infestation could play an important role in the dissemination of screwworm. The home range of a feral swine can be over 400 ha, and their territorial range is not limited by rivers [35]. In fact, some of the hunters taking part in the present study reported that during some of the hunting operations in parts of Artigas Department where the Cuareim river serves as the international boundary, feral swine escaped capture by jumping and swimming to the bank of the river on the Brazilian side of the border. It is probable that screwworm infestations acquired in Uruguay, Argentina, or Brazil could be carried to a bordering country where the larvae would crawl off and pupate. This represents a potential for a transboundary zoonotic disease issue. The results from this study highlight the ability of feral swine to act as host for screwworm populations in areas where both are endemic. If one of these three countries were to begin a control or eradication program, feral swine would be a source for re-infestation.

Cooperation with local hunters through ProJAB enabled screwworm infestation to be assessed in feral swine in this study, stressing how research and extension efforts facilitate collaboration between groups that deal with issues at the livestock–wildlife interface. This public–private partnership also involves the education of hunters on practical aspects of veterinary public health to mitigate risks associated with exposure to zoonoses harbored by feral swine in Uruguay [20]. Hunters were made aware of measures to avoid the dispersal of screwworms and to manage the risk of human and domestic animal exposure to infestation. Hunting feral swine often occurs at night, and the hunters transport the harvested animals to their homes and leave them hanging until morning when they are cleaned and processed. During this time, the third-instar larvae would be able to crawl off and pupate near the homes of the hunters, increasing the presence of screwworm adults in the vicinity of the hunters’ homes. In Uruguay, up to 818 human cases of screwworm myiasis are recorded annually, affecting mainly rural populations [24]. This stresses the need to address the control measures of screwworm myiasis from a One Health perspective, especially since it is a neglected zoonosis in the region [36].

The ecological classification of myiasis describes screwworm as an obligate primary parasite because it is dependent on a living host and is capable of initiating the myiasis [37]. Secondary infestations, whether facultative or accidental, with myiasis-causing species in the families Calliphoridae, Sarcophagidae, and Muscidae are often associated with established screwworm infestations [37, 38]. In myiases with secondary species, the primary screwworm *C. hominivorax* is found feeding on living tissues, while the secondary species are at the wound periphery consuming necrotic tissues. All of the larvae collected from the myiasis cases in this study were identified as *C. hominivorax*, with no secondary myiasis-causing species present. The absence of secondary fly larvae in these infested feral swine could be due to the wallowing and swimming behaviors mentioned previously and account for the low reported mortality associated with myiases. If myiases are not persistent and tissues do not become necrotic, the development of secondary infestation could be less likely.

Seasonal changes in temperature were correlated with screwworm prevalence in southern Texas in cattle, sheep, and other livestock prior to eradication [39]. However, the patterns are dependent on local climates and should not be generalized. Screwworm infestation in this study correlated with higher monthly average temperatures, and fewer cases were detected in winter months with low temperatures. An increase in infestations associated with higher temperature is concerning because it has been estimated that current global change trends could result in an increase in temperatures in Uruguay of up to 3 °C by 2100 [40]. Under this scenario for other parts of the Americas [41], the risk for screwworm infestation could extend further into the year, thereby reducing the efficacy of seasonal birthing currently practiced by livestock producers in parts of Uruguay with a more temperate climate to reduce screwworm cases. In our study, total monthly precipitation was not correlated to myiases prevalence, as has been seen in screwworm surveys in tropical countries such as Panama or the Caribbean [42, 43]. However, the risk for screwworm outbreaks where domestic pigs and feral swine are present must be noted. Two pet pigs were infested during the 2016 outbreak in Florida, and feral swine also thrive in Panama where the screwworm barrier zone exists to prevent the reinvasion of Central and North America through the continental mainland [6, 44].

## Conclusions

Efforts to manage screwworm populations need to include surveillance of myiasis in feral swine where these two high-consequence pests of zoonotic importance coexist. Feral swine in Uruguay were documented to be a common screwworm host. Additionally, feral swine populations are growing and becoming established in new areas, possibly providing additional suitable hosts for screwworm. Moreover, feral swine appear to be resilient to screwworm infestation, unlike the Key deer in southern Florida. Although precautions are taken to reduce screwworm cases in livestock, the invasive feral swine in Uruguay are common hosts to screwworm and possibly serve as a source of infestation for livestock and humans. This situation and the public–private partnership with hunters in Uruguay that facilitated this research project emphasize the relevance of taking the One Health approach to deal with invasive species and transboundary zoonotic diseases.

## Supplementary information

**Additional file 1: Dataset S1.** Data for all feral swine collected in the study. Data contains: date of sample, sex and size of captured feral swine, coordinates of capture, and if the swine had myiasis.

**Additional file 2: Dataset S2.** All weather data used for correlations with temperature and precipitation.

**Additional file 3: Dataset S3.** Data for all myiasis cases. GPS coordinates, location of sample, body location of myiasis, reported probable cause of myiasis.

## Data Availability

All data generated or analyzed during this study are included in this published article and its additional information files.

## Abbreviations

ACJA: Asociación de Controladores de Jabalí de Artigas
ProJAB: Proyecto Jabalí
